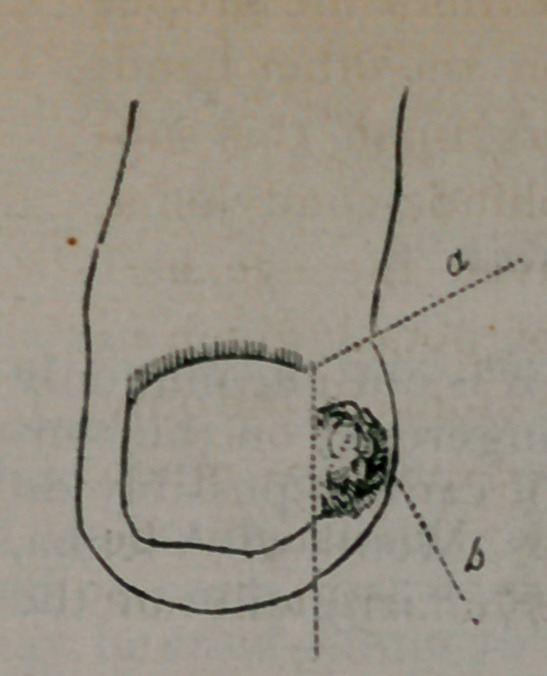# Ingrowing Toe-Nail

**Published:** 1883-01-20

**Authors:** R. C. Word

**Affiliations:** Professor of Physiology in the Southern Medical College, Atlanta, Ga.


### INGROWING TOE-NAIL.

By R. C. Word, M.D.,

Professor of Physiology in Southern Medical College, Atlanta, Ga.

Perhaps the most annoying trouble encountered in minor surgery is the ingrowing toe-nail. Its apparent insignificance makes the difficulty of relieving it the more provoking.

The operative methods which have been resorted to are numerous, but none yet devised are wholly satisfactory.

The tearing away of a part or the whole of the nail, as usually practiced, is an exceedingly painful operation, and seems altogether out of proportion to the trivial character of the cause of the trouble, and the nail, not unfrequently, returns after its removal with the same malgrowth and a renewal of the former suffering.

Three months ago I devised and performed a comparatively painless and simple operation for an obstinate ingrowing nail, in the case of a lad of fifteen years of age, which had existed for two years, and upon which all the usual temporizing methods had been unsuccessfully tried. Having allowed ample time to test the result of the operation and finding it wholly satisfactory and successful, I here publish it for the benefit of the profession.

It consists in removing the flesh, with a very small portion of the toe nail, from the affected side, by an incision commencing from a point a little above and including a portion of the root of the nail, as seen in the accompanying cut.

The letter *b* shows the swollen part of the toe and the exhuberant granulations springing from the ingrowing point. The letter *a* shows the line of incision entering obliquely at a point a little above the upper corner or angle of the nail and passing downward, as seen in the dotted line, the angle of incision being just above the matrix or root of the nail. The margin at the upper part should have been represented in the cut as passing a little beyond the angle of incision.

The instrument used was a very narrow bistoury (a small, short pen blade will answer). It should be narrow, so as to make convenient to the operator the angle or turn for the downward cut, which is best made continuously from above downward, like the trimming of the side of a goose-quill pen. Let the ball of the toe below be made taut by grasping it between the thumb and finger as the incision is made, and the entire flesh on the affected side be cut away, including a strip from the side of the nail about two lines in width, with a portion of the root above.

In many cases, doubtless, it would be unnecessary to cut away any portion of the nail, could we know its exact condition, but as there are cases in which the inward curvature is considerable, and sometimes a hidden detached spiculum of nail penetrating the toe, it were better to provide against all contingencies of the kind and make a sure thing of the first incision. A simple dressing with lint and bandage suffices to stop the bleeding. This may be removed four or five days afterward by softening with tepid water, and the wound will heal rapidly. In the above case new skin completely covered the wound in about two weeks, the toe presenting a somewhat narrowed appearance, and in three months the nail has fully grown out, sound and natural in appearance. There now exists no indication or probability of any future return of the trouble.

## Figures and Tables

**Figure f1:**